# Mesenchymal chondrosarcoma of the chest wall in an adolescent patient: A case report and brief review of the literature

**DOI:** 10.1002/cnr2.1453

**Published:** 2021-06-16

**Authors:** Marti Goldenberg, Archana (AR) Ramgopal, Cláudia M. Salgado, Miguel Reyes‐Múgica, Marcus M. Malek, Jean M. Tersak

**Affiliations:** ^1^ UPMC Children's Hospital of Pittsburgh University of Pittsburgh Pittsburgh Pennsylvania USA

**Keywords:** chest wall, extraskeletal, mediastinum, mesenchymal chondrosarcoma, tumor

## Abstract

**Background:**

Mesenchymal chondrosarcoma is a rare and aggressive bone tumor with few reports of primary tumor in the chest wall.

**Case:**

We report a case of a 17‐year‐old male presenting with back pain and a posterior mediastinal mass. Imaging demonstrated what was thought to be a benign chondral tumor. The patient underwent resection which confirmed extraskeletal mesenchymal chondrosarcoma. The patient declined proposed adjuvant chemotherapy and underwent multiple resections for rapid local reoccurrence. He ultimately elected for hospice care.

**Conclusion:**

The case highlights the importance of close disease monitoring and exploration of treatment options, given lack of established guidelines and consistent tumor features.

**Abbreviations**
CT: Computed tomography
ED: Emergency department
MCS: Mesenchymal chondrosarcoma
MRI: Magnetic resonance imaging

## 1 INTRODUCTION

Mesenchymal chondrosarcoma (MCS) is a rare malignancy with significant morbidity and mortality given, its aggressive features and high likelihood for delayed distant metastasis with late recurrence. We report a case of incompletely treated MCS and discuss the utility of early detection and imaging, given the rapid rate of local recurrence without systemic therapy.

## 2 CASE REPORT

**FIGURE 1:**
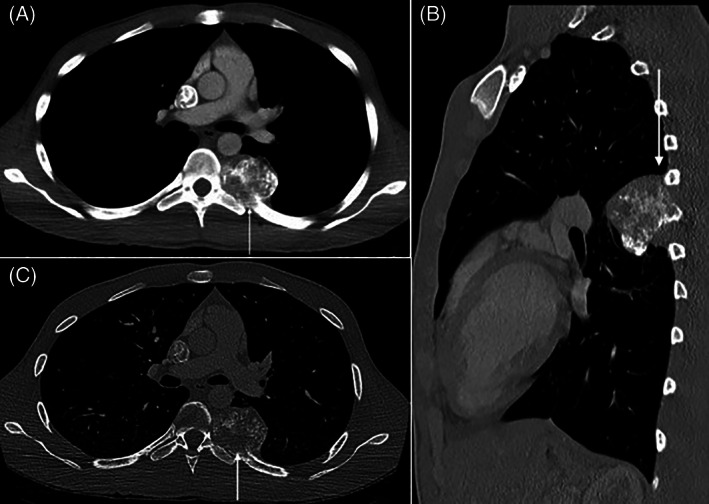
Initial axial contrast–enhanced chest CT mediastinal window (A), axial (B) and sagittal l bone window (C) images demonstrates a 5 cm mass arising from posterior left seventh rib containing cloud like calcification (white arrow) without spinal canal or neural foraminal extension

A 17‐year‐old healthy male presented to the emergency department (ED) for worsening chronic left‐sided back pain with intermittent numbness, inspiratory pain, and a 30‐pound weight loss. He had a left lung calcification on a chest film taken 8 months prior to admission, which was thought to be benign calcium deposition. Tuberculosis quantiferon gold and human immunodeficiency virus antibody titers were undetectable. A computed tomography (CT) scan demonstrated a single 4 × 5 × 5 cm posterior mediastinal mass with destruction of the posterior aspect of the left seventh rib (Figure 1).

**FIGURE 2:**
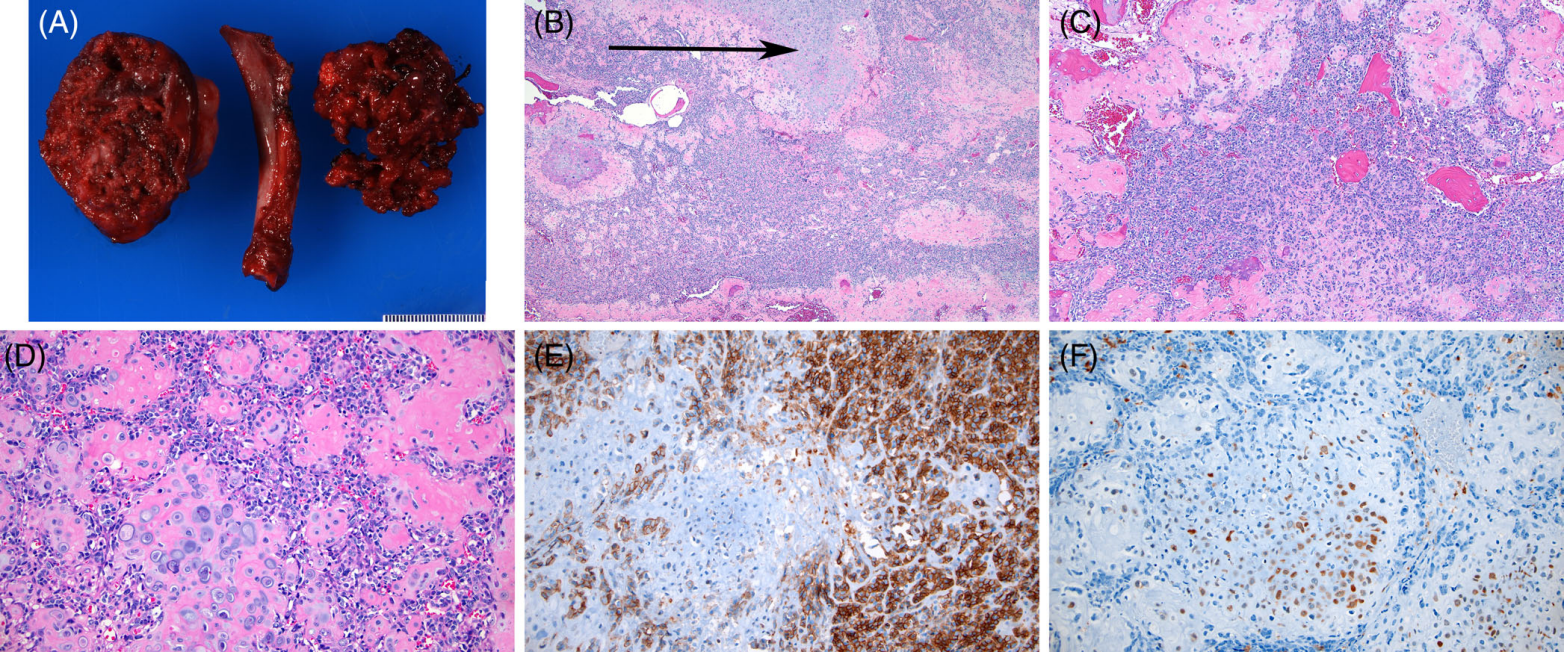
Representative images of the mesenchymal chondrosarcoma resected from the thorax and left rib. (A) Macroscopic image showing a 6 × 5.5 × 3 cm mass on the left, a segment of the resected rib in the middle, and an additional portion of fragmented tumor. Note the irregular and variegated appearance of the cut surface of the tumor (left). (B) Low‐power view of the classic biphasic pattern in mesenchymal chondrosarcoma. Small, “blue” round cells alternate with cartilaginous nodules (HE; 40X). (C) The small cell component predominates in this field and infiltrates between nodules of cartilage (HE 100X). (D) Close‐up of a central nodule of neoplastic cartilage surrounded by the infiltrating small‐cell component. Several small nodules of cartilage are seen between the small cells (HE; 200X). (E) CD99 immunohistochemistry shows a strong membranous staining pattern in the small cell component of chondromesenchymal chondrosarcoma (CD99; 200X). (F) The cartilaginous component is highlighted by S100 immunohistochemical staining (S100; 200X)

Magnetic resonance imaging (MRI) of the cervical and thoracic spine confirmed CT findings, without neurological deficits on examination. A percutaneous biopsy revealed an atypical chondroid lesion consistent with a sessile osteochondroma. Given the continued significant pain, he was taken to the operating room for resection of the presumed benign lesion. Intraoperatively, it was noted to be an aggressive lesion, with bony destruction. Pathology review demonstrated MCS with both primitive small‐cell and bland cartilaginous components, with positive margins (Figure 2). A sarcoma fusion NGS panel analysis detected a HEY1‐NCOA2 fusion.

Repeat CT scan showed postoperative changes to the left posterior mediastinum with residual amorphous density and no lymphadenopathy. There was mild flouro‐deoxyglucose uptake on positron emission tomography scan of the chest.

**FIGURE 3:**
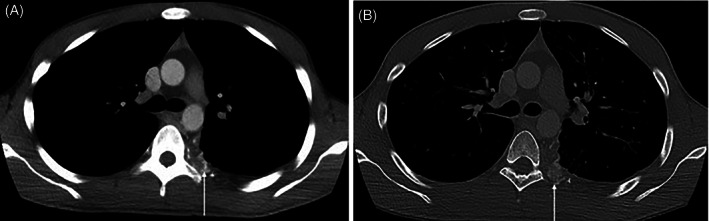
Two‐month follow‐up axial contrast enhanced chest CT mediastinal window image (A), and bone window (B) demonstrates amorphous calcification (white arrow) at postoperative site in left T7 level

An adjuvant chemotherapeutic plan of doxorubicin, ifosfamide, and dexrazoxane for four 21‐day cycles, followed by resection with attempted negative margins, was proposed to the patient, per an Ewing sarcoma–backbone chemotherapy regimen.^1^ Additionally, radiation was offered and declined. The patient chose repeat tumor resection to attempt to achieve negative margins, in lieu of chemotherapy. Two months after initial excisional biopsy, a repeat wide re‐excision via left thoracotomy with multiple rib disarticulations was performed (Figure 3). Pathology review showed extension of the tumor periosteally, superiorly, and focally to medial resection margins without lymph node involvement. The second specimen had a greater portion of small‐cell tumor relative to bland cartilaginous components with positive margins. Despite our team's recommendations, the patient declined all further treatment‐directed interventions.

**FIGURE 4:**
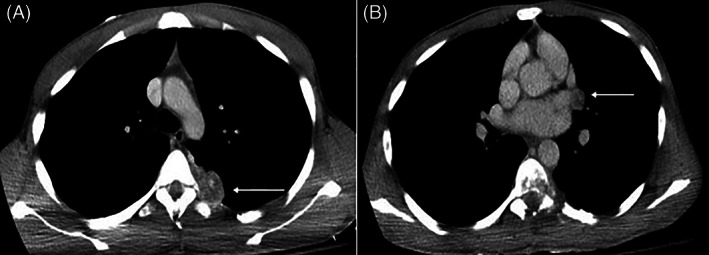
Eleven‐month follow‐up axial contrast enhanced chest CT mediastinal window at T7 level (A); white arrow demonstrates interval progression of residual tumor and left perihilar lymphadenopathy (B)

The patient presented 5 months later to the ED with lower extremity weakness and paresthesia. Physical exam was significant for decreased lower extremity strength with clonus and hyperreflexia. MRI of total spine revealed local tumor recurrence at T6‐7 with cord compression and interval increase in sub‐pleural lesions. He was taken for an emergent laminectomy of T6‐7 followed by a post‐surgical steroid taper. Histology showed biphasic morphology consistent with prior masses. Follow‐up CT scan was notable for multiple left anterior paravertebral/pleural masses with peri‐hilar and costophrenic lymphadenopathy (Figure 4). His lower extremity weakness improved, and the patient ultimately elected to pursue hospice care which he has been on for 6 months.

## 3 DISCUSSION

MCS is a highly heterogeneous tumor first described by Lichtenstein and Bernstein (1959) which accounts for less than 10% of all chondrosarcomas.^1^, ^2^ It has a biphasic pattern of undifferentiated small round cells intermixed with islands of well‐differentiated cartilage.^3^ This rare tumor can arise from skeletal or extraskeletal origins, a feature that defines it from conventional chondrosarcoma.^4^ Extraskeletal MCS are now thought to comprise up to 50% of total MCS and arise most commonly in the central nervous system and meninges but also have been identified in the lower extremity, head and neck, and axial soft tissues.^4^, ^5^, ^6^, ^8^ MCS has no sex predilection and arises during the second and third decades of life.^3^, ^9^

The presence of undifferentiated small round cells, positive for CD99 immunohistochemistry in a membranous pattern, and also positive for NKX2.2 in a nuclear pattern may represent a pitfall since Ewing sarcoma (EWS), another relatively frequent primary bone tumor, is also positive for these markers.^3^, ^6a^ However, MCS differs from EWS as the only bone tumor with a component of developed cartilage, which stains positive for the S‐100 protein.^3^, ^8^ Recent studies have identified a novel fusion gene HEY1‐NCOA2 which has both high sensitivity and specificity for MCS.^10^ Detection of this fusion gene may play a role in future cytogenetic diagnosis of obscure presentations of MCS.^1^

Patients most commonly present with a palpable mass, swelling, or pain.^11^ Progressive neurological deficits can also be seen with involvement of the central nervous system, meninges, and in progressive disease.^12^ The limited number of case reports with MCS of the mediastinum describes nonspecific presenting symptoms including inspiratory pain, cough, and shortness of breath.^13^, ^14^ Intraspinal tumors have associated back pain similar to this patient; however, these may have focal neurologic deficits relative to spinal level.^4^, ^15^

There are no hallmark features on imaging, which renders the importance of histology for diagnosis.^1^ One may observe calcifications or osteolytic features that mimic conventional chondrosarcomas.^15^ Likewise, biopsy may not yield a clear diagnosis because the tumor has multiple components, as was in this case report. For that reason, clinicians should consider the possibility of a nonrepresentative biopsy if symptoms persist or the tumor progresses.

MCS is associated with a poor prognosis and a highly protracted course. Overall survival estimates are variable across studies (20–67%), likely attributable to the rarity of the tumor and small sample sizes in each individual study.^5^, ^9^, ^16^, ^17^ Likewise, there are no definitive predictive factors.^9^, ^17^ Possible prognostic factors include younger median age of diagnosis, anatomic location of tumor, presence of metastasis, and time at detectable recurrence.^9^, ^16^, ^17^, ^18^ Schneiderman et al found individuals with MCS of the head and neck to have better survival outcomes, perhaps secondary to early detection. This conclusion is echoed in other studies which determined superficial tumor location to be important to overall outcomes and early detection.^11^

MCS has a propensity for late local recurrence in addition to distant metastasis, most commonly to the bone and lungs.^2^, ^3^, ^6^, ^8^, ^16^ Studies have detected metastasis at 20+ years following initial treatment of the primary tumor warranting long‐term follow‐up for all patients regardless of initial presentation.^1^, ^3^, ^8^, ^9^

Radical wide excision with clear margins is the mainstay of treatment.^8^, ^9^, ^11^ Chemotherapy and radiation are pursued if the primary tumor is not surgically resectable which is important, given that most chondrosarcomas are surgically corrected alone.^11^ Adjuvant chemotherapy and radiation are controversial.^1^, ^9^, ^16^, ^17^, ^18^ Frezza at al showed a significant reduction in disease recurrence with adjuvant chemotherapy which was reflected in other studies.^17^ Chemotherapeutic agents used are the same backbone as those used to treat EWS, given common tumor characteristics of highly malignant behavior, younger age, and involvement of nonosseous tissues.^1^, ^16^

## 4 CONCLUSIONS

This is a unique presentation of extraskeletal MCS within the chest wall of an adolescent patient that had rapid rate of local recurrence in the absence of systemic treatment. In agreement with the literature, this case demonstrates the importance of complete histological examination of the resected mass in diagnosing MCS and highlights that there are no consistent symptoms or imaging features for clinical diagnosis. The HEY1‐NCOA2 fusion gene detected in this case is considered currently confirmatory of the diagnosis of MCS. Further discovery is warranted on the use of genetic analyses and associated clinical behavior of these tumors, including anticipated time frames for recurrence and metastasis. Finally, there is no clinical consensus on adjuvant chemotherapeutic and radiation treatment as studies report conflicting information on outcomes. Longitudinal follow‐up studies will be beneficial for establishing treatment guidelines in addition to recording specific chemotherapeutic agents successful in treating MCS.

## AUTHOR CONTRIBUTIONS

**Marti Goldenberg:** Conceptualization; investigation; methodology; project administration; supervision; visualization; writing‐original draft; writing‐review & editing. **Archana (AR) Ramgopal:** Conceptualization; investigation; methodology; project administration; supervision; visualization; writing‐original draft; writing‐review & editing. **Caludia Salgado:** Investigation; methodology; visualization; writing‐original draft; writing‐review & editing. **Miguel Reyes‐Mugica:** Investigation; methodology; visualization; writing‐original draft; writing‐review & editing. **Marcus Malek:** Conceptualization; investigation; methodology; project administration; supervision; visualization; writing‐original draft; writing‐review & editing. **Jean Tersak:** Conceptualization; investigation; methodology; project administration; visualization; writing‐original draft; writing‐review & editing.

## CONFLICT OF INTEREST

All authors have no conflicts of interest to disclose.

## ETHICAL STATEMENT

This report is anonymized to protect the patient described. This study does not require approval by the Institutional Ethical Committee, and the patient was notified of the published report.

## FINANCIAL DISCLOSURE

All authors have no financial relationships relevant to this article to disclose.

## Data Availability

Data sharing not applicable to this article as no datasets were generated or analyzed during the current study.
